# Impact of Milk Storage and Heat Treatments on In Vitro Protein Digestibility of Soft Cheese

**DOI:** 10.3390/foods12081735

**Published:** 2023-04-21

**Authors:** Simona Rinaldi, Sabrina Di Giovanni, Giuliano Palocci, Michela Contò, Roberto Steri, Carmela Tripaldi

**Affiliations:** Consiglio per la Ricerca in Agricoltura e l’Analisi dell’Economia Agraria (CREA), Centro di Ricerca Zootecnia e Acquacoltura, Via Salaria, 31, 00015 Rome, Italy; sabrina.digiovanni@libero.it (S.D.G.); giuliano.palocci@crea.gov.it (G.P.); michela.conto@crea.gov.it (M.C.); roberto.steri@crea.gov.it (R.S.); carmela.tripaldi@crea.gov.it (C.T.)

**Keywords:** digestion, protein, peptides, accessibility, amino acids

## Abstract

Cheese is an important source of protein in the human diet, and its digestibility depends on its macro and microstructure. This study investigated the effect of milk heat pre-treatment and pasteurization level on the protein digestibility of produced cheese. An in vitro digestion method was used considering cheeses after 4 and 21 days of storage. The peptide profile and amino acids (AAs) released in digestion were analyzed to evaluate the level of protein degradation following in vitro digestion. The results showed the presence of shorter peptides in the digested cheese from pre-treated milk and 4-day ripening while this trend was not observed after 21 days of storage, showing the effect of storage period. A significantly higher content of AAs was found in digested cheese produced from milk subjected to a higher temperature of pasteurization, and there was a significant increase in total AA content in the cheese after 21 days of storage, confirming the positive effect of ripening on protein digestibility. From these results emerges the importance of the management of heat treatments on the digestion of proteins in soft cheese.

## 1. Introduction

The importance of protein intake in the human diet is unquestionable, and various dairy products such as liquid milk, yoghurt, and cheese, are excellent sources of animal proteins [1]. Dairy products supply high-quality proteins both for their content of essential amino acids and their high digestibility [2]. Moreover, dairy proteins are recognized to contain bioactive peptides which can be released during gastrointestinal digestion [3].

Milk proteins are components of different matrices from liquid milk, fermented milk, and soft or hard cheeses, and therefore, protein digestion kinetics depend on the matrix in which the protein is located [4]. The dairy food matrix has shown a relevant impact on bioaccessibility (nutrient release in the intestinal lumen) and subsequent nutrient bioavailability because nutrients need to be released from the food matrix during digestion for their absorption [5,6,7]. During processing, dairy products are subject to different conditions of treatment (thermal, enzymatic, acid and mechanical) that induce modifications in milk proteins [7,8]. Cheese is a complex matrix consisting of a casein network whose alveoli are occupied by fat and serum. Each cheese type has an expected dominant texture, induced by different manufacturing steps and compositions [9]. Thus, the cheese matrix may affect the kinetics of protein digestion, influencing amino acid release and its subsequent adsorption in plasma [10].

Heat treatments are widely used during dairy processing to ensure food safety by inhibiting microbial growth, extending products’ shelf life, and improving product quality. The most common heat treatments applied to milk are pasteurization, which consists of heating to a minimum of 73 °C for 15 s or equivalent parameters, and ultra-high temperature (UHT) sterilization, involving heating at 135–150 °C for 1–2 s [6]. The main modifications induced by thermal treatments are denaturation and aggregation of proteins, and their consequences depend on the severity of the heat treatment (temperature and time) [2]. When milk is heated at temperatures above 70 °C, the whey proteins, which mainly include α-lactalbumin (α-La) and β-lactoglobulin (β-Lg), are denatured by unfolding and the resulting exposure of previously buried hydrophobic areas [11]. On the contrary, the casein structure is stable because it is associated in the form of casein micelles, and individual casein proteins are not subjected to denaturation upon heating. Caseins are, however, sensitive to processing that induces chemical modifications and the associated aggregation [12]. The whey proteins form aggregates upon heating and also aggregate with casein micelles, due mainly to disulfide bridge formation. In particular, k-casein, located on the surface of the micelle, forms complexes with denatured serum proteins [6].

Altogether, protein modifications may be considered indicative of processing severity, and these changes in the food matrix could induce physico-chemical interactions that could improve or worsen the bioaccessibility of nutrients. Protein denaturation can also alter the gastric emptying of the protein, consequently affecting digestive kinetics [2]. Typical methods used to study protein digestion include in vitro methods. Although in vitro methods do not always match what happens in vivo, due to the difficulty of simulating the complexity of the digestive tract and its related events, in vitro digestion models are widely used to study the digestive behavior of foods as they are faster and cheaper than in vivo methods, and devoid of ethical implications [13].

In a previous study that was recently published [14], we evaluated the effect of milk storage time and heat treatments on the chemical and oxidative characteristics and proteolysis levels of cheese. In the present work, the objective is to investigate the combined effect of milk storage and milk heat treatments on the digestibility of soft cheese. For this purpose, an in vitro digestion method was used considering fresh cheese (after 4 days of storage) and shortly aged cheese (after 21 days of storage). The peptide profile and amino acid content were analyzed in the digested samples to evaluate the level of protein degradation and amino acid release after in vitro digestion.

We hypothesized that the storage and heat treatment of milk could influence the protein digestibility of the soft cheese produced. Increasing the digestibility of cheeses is a topic of great interest for consumers, particularly children, the elderly and those in the presence of special nutritional needs. We therefore want to evaluate whether the use of fresh milk affects the digestibility of cheese protein and whether or not the intensity of the heat treatments (pre-treatment and/or pasteurization temperature) could in some way modulate its digestibility.

## 2. Materials and Methods

### 2.1. Cheese Production

The experimental cheese-making trials were carried out at the small experimental dairy plant of CREA (Bella Muro, Potenza, Italy). The cheese was produced with cow’s milk collected from local farms, stored at 4 °C and transferred to the experimental dairy plant within 24 h of the first milking. The received milk was subjected to two different types of storage:

- Fresh milk (F) was processed within 48 h of the first milking,
- Pre-treated stored milk (P) was thermized at 65 °C for 15 s upon receipt (within about 24 h of the first milking) and processed within 96 h of the first milking.

In the dairy plant, the milk was stored in tanks at 4 °C, and before processing, the milk was pasteurized using a plate exchanger (Comat Dairy Equipment, Bellizzi, Salerno, Italy) equipped with software for temperature and time control. Pasteurization was performed at two temperature levels:

- At 72 °C for 15 s, called minimal pasteurization (M), the minimum recommended level for milk pasteurization,
- At 77 °C for 15 s, called strong pasteurization (S), a higher-temperature condition of pasteurization to test the effect on produced cheese.

The cheese was produced artisanally following the protocol detailed by Tripaldi et al. [15]. Four different kinds of cheese were produced from the four treated milk (F-M, F-S, P-M, and P-S). For each kind of cheese, the molds were carried to the laboratory of CREA (Monterotondo, Rome, Italy), stored at 4 °C and half of them were analyzed after 4 days of storage for the study of fresh cheese, while the other half were analyzed after 21 days for the study of shortly aged cheese.

The experimental plan included two types of milk storage (F and P) subjected to two pasteurization levels (M and S) and two periods of cheese storage (4 days and 21 days), for three repetitions (2 milk types × 2 pasteurization levels × 2 cheese storage × 3 repetitions).

The pH was determined for the whole mold, and then the cheeses were homogenized without separating the core from the surface, divided into sub-samples and frozen at −80 °C until analysis.

### 2.2. Physico-Chemical Analyses of Cheeses

The cheese samples were analyzed using the following standard indicators to determine the main chemical characteristics: pH, moisture (IDF 1986) [16], ash (AOAC 2000) [17], and protein and fat content (ISO 21543 IDF 201 2006) [18]. 

### 2.3. In Vitro Digestion Model

To obtain the digested samples, cheeses from different milk treatments at 4 and 21 days of storage were subjected to in vitro digestion. The digestion process involves three steps simulating digestive processes in the mouth, stomach, and small intestine. In vitro digestion was based on the cheese model of Fang et al. [9] with several modifications. The content of enzymes added at each digestion step was reported by Rinaldi et al. [4], and the digestive juices were replaced with a buffer solution (120 mM NaCl, 5 mM KCl, and 6 mM CaCl_2_, at a pH of 6.9) as reported by Bordoni et al. [19]. All the enzymes used were purchased from Sigma-Aldrich (Oakville, ON, Canada), and these were α-amylase (A3176), pepsin (P7000), lipase (L3126), bile (B8631) and pancreatin (P7545).

In vitro digestion was realized in a beaker kept at 37 °C using a water bath on a magnetic stirrer, to ensure a good dispersion of cheese particles, equipped with a heating plate. Digestion began by adding 6 mL of a buffer solution, in which α-amylase was previously dissolved, to the lyophilized cheese sample (9 g) and incubating it for 2 min. For the gastric digestion phase, 10 mL of a buffer solution with pepsin was added to the beaker and the mixture was agitated. Another 2 mL of the buffer with pepsin was further added after an hour of gastric digestion. During this phase of digestion, the pH was maintained at 2–3 with HCl to simulate conditions in vivo.

After two hours of incubation, at the end of gastric digestion, the pH was adjusted to 6–7 by NaHCO_3_. Then, 18 mL of a buffer solution with pancreatin, lipase and bile and 2 mL of a bicarbonate solution were added to start the duodenal digestion that lasted 3 h in the same agitation condition. The temperature during the whole digestion was maintained at 37 ± 1 °C. 

At the end of digestion, the digested samples were neutralized with NaOH/HCl to reach a pH of 7.00 ± 0.05. To stop the enzymatic digestion, the digested samples were heat treated at 90 °C for 10 min and cooled in ice for 10 min. All samples were centrifuged at 9800× *g* for 20 min (4 °C), obtaining an upper layer composed mainly of fat, supernatant, and pellet. After removing the surface fat layer, the supernatant was filtered with 0.2 μm membranes while the fat layer and the pellet were not collected. 

The supernatant obtained after the centrifugation contained the in vitro digestion products released into the solution following liberation from the matrix.

An aliquot of the supernatant was sequentially ultrafiltered with Amicon Ultra (Millipore, Bedford, MA, USA) at 30 kDa of the molecular weight cut-off. For each sample collected during the digestion procedure, two aliquots were finally obtained: a fraction containing molecules with a molar mass of >30 kDa and a fraction containing molecules with a molar mass of <30 kDa that was defined as the digested sample.

The digested sample containing solubilized molecules with a molecular mass of <30 kDa represented the fraction with the most degraded compounds released in the solution from the food matrix, and for this reason, was more suitable to subsequent catabolism and uptake at the intestinal level. The digested samples (<30 kDa) were kept frozen at −80 °C until their analysis. 

### 2.4. Peptide Profile Analysis before and after Digestion

The peptide profile of the cheeses before digestion was analyzed following the sample preparation procedure described by Meira et al. [20]. The peptide profile in both the undigested and digested samples was determined by HPLC analysis, in accordance with the method of Parrot et al. [21]. 

A Waters Alliance 2695 HPLC system (Waters, Milford, MA, USA) equipped with an ACME C18 column (250 × 3.0 mm, 5 μm, 120 A, Phase Analytical Technology, LLC, State College, PA) and a pre-column packed with the same stationary phase, was used. Samples (20 μL) were loaded onto the column and eluted for 5 min with solvent A, Milli-Q water with 0.1% (*v*/*v*) trifluoroacetic acid (TFA), and solvent B, acetonitrile with 0.1% (*v*/*v*) TFA at a 90:10 *v*/*v* rate. Peptides were then eluted by a linear gradient from 10 to 50% *v/v* of solvent B for 45 min followed by a second linear gradient from 50 to 10% *v/v* of solvent B for 10 min to restore the initial conditions. The flow rate was 1 mL min^−1^, the oven temperature was 40 °C, and detection was carried out at 214 nm. 

The mean retention time was calculated as described by Parrot et al. [21] to evaluate the retention time distribution of the peaks. First, the area of each interval was multiplied by the mean retention time of the corresponding interval, summed up and then divided by the total peak areas.

### 2.5. Analysis of Amino Acids

The amino acid (AA) content in the digested samples was detected using the AccQ•Tag Flour reagent kit by Waters (Milford, MA, USA) in Waters Alliance 2695 HPLC on a 3.9 × 150 mm AccQ•Tag column (Waters, Milford, MA, USA). The samples were derivatized with an AccQ•Tag reagent and 10 μL was injected. Amino acids (AAs) were detected by fluorescence at EX = 205 nm, EM = 395 nm with a mobile phase gradient of Eluent AccQ•Tag: Acetonitrile: Ultrapure water (*v*/*v*/*v*) and an oven temperature at 37 °C.

Individual AAs were identified according to their retention times by comparison with the chromatogram of a standard solution mixture of 18 amino acids (Waters, Milford, MA, USA), and each peak was integrated. The concentration of each AA was determined, and the amount of all AAs (ΣAAs) was calculated to evaluate the total AAs contained in the digested sample.

### 2.6. Statistical Analysis

In order to investigate the effect of the type of milk and milk heat treatments on the digestibility of the cheese processed, all data were analyzed using the general linear model (GLM) procedure in the SAS software version 9.3 (SAS Inst. Inc., Cary, NC, USA) [22]. The model included three fixed factors, with two levels each: type of milk (F and P), pasteurization level (M and S) and cheese time storage (4 days and 21 days), also considering their interactions. Differences between the least square means were considered significant at *p* < 0.05 using Tukey’s test.

A principal component analysis (PCA) was performed on a total of 29 variables (physico-chemical characteristics, peptide profiles after digestion and AA content), in order to identify the underlying structures in the data set related to the different experimental theses. Scores for each sample were calculated and plotted in the extracted principal component space. The evaluation of the loadings of each eigenvector was used to infer the underlying biological mechanisms related to heat treatment and storage. PCA was performed by the PROC PRINCOMP procedure of SAS [22].

## 3. Results and Discussion

### 3.1. Characteristics of Cheese

The main physico-chemical characteristics of the cheese produced using fresh and pre-treated milk subjected to minimal or strong pasteurization are shown in Table 1. The mean values of cheese characteristics were reported considering the three fixed factors, with two levels each: type of milk (F and P), pasteurization level (M and S) and cheese time storage (4 days and 21 days), also considering their interactions. The non-significant m*s and p*s interactions suggested that the 4-day and 21-day cheeses were affected in the same way by the other factors, i.e., the effect of milk treatment did not depend on the aging time.

**Figure 1:**
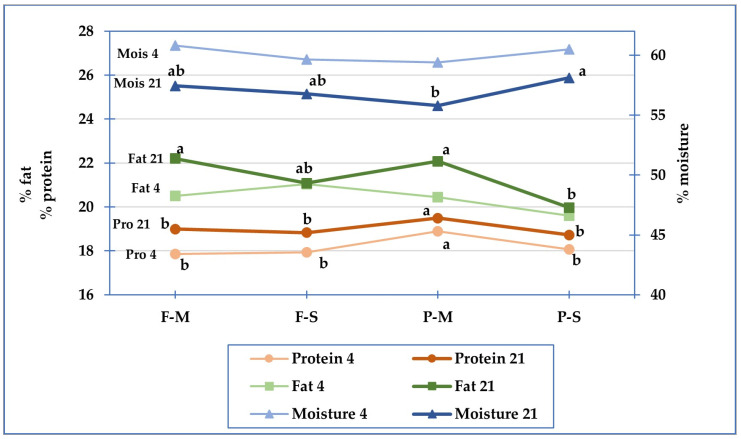
Moisture (Mois), protein (Pro) and fat content in cheese after 4 and 21 days of storage, made from the four different types of milk. F-M: fresh minimally pasteurized milk; F-S: fresh strongly pasteurized milk; P-M: pre-treated minimally pasteurized milk; P-S: pre-treated strongly pasteurized milk. For each parameter, different letters in the graph indicate significant differences (*p* < 0.05).

On the contrary, there was an interaction between milk and pasteurization level (m*p, Table 1) for the moisture and protein content in the cheese, indicating that the two factors together had different effects in the different combinations of treatments (F-M, F-S, P-M, and P-S). To show the different trends of moisture and protein content, we reported these parameters in Figure 1 considering the cheese obtained from the four different types of milk after 4 and 21 days of cheese storage, separately. Additionally, the trend of fat content is shown in the graph.

The data reported in the graph show an inverse correlation between moisture and protein content, so in the pre-treated lowly pasteurized milk cheese (P-M), the reduction in moisture in the cheese led to an increase in protein content. In particular, the 21-day-aged P-M cheese showed a decrease in moisture % compared to P-S. An interaction between pre-treatment and the minimal pasteurization of milk resulted in less water retention in the cheese. Instead, in the cheese from pre-treated strongly pasteurized milk (P-S), the increase in the pasteurization temperature of the milk increased water retention and consequently decreased protein content. Higher heat treatments caused a weakening of the texture of the curd, which retained a greater amount of water as a paracasein–whey protein complex has a greater ability to bind water [23].

### 3.2. Peptide Profile before and after In Vitro Digestion

**Figure 2:**
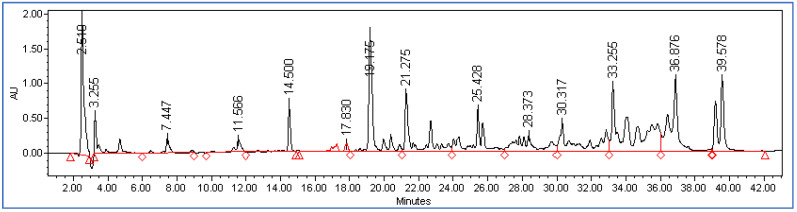
Chromatographic example of a peptide profile of an undigested cheese.

**Figure 3:**
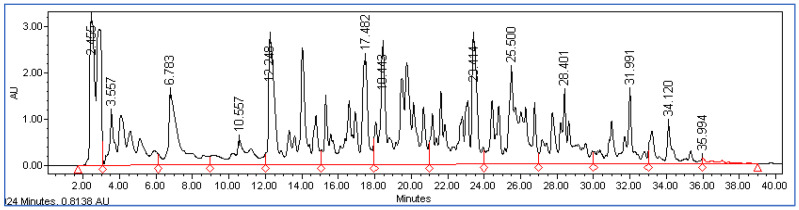
Chromatographic example of a peptide profile of a digested cheese.

The peptide profile was determined for both the undigested and digested cheeses after 4 and 21 days of storage for all the milk treatments. The chromatograms obtained were divided into different sections considering four retention time intervals, the area of each section was normalized with respect to the total area and the mean retention time was calculated. Figure 2 and Figure 3 show a chromatographic example of a peptide profile of an undigested cheese and a digested cheese, respectively.

To describe the peptide profile, the area % of each section, the total area and the mean retention time are shown in Table 2 for the undigested cheese and in Table 3 for the digested cheese, considering the three factors: milk, pasteurization level and cheese storage.

The analysis of the peptide profile did not give information on the single peptides present in the sample, but it allowed the characterization of each cheese, indicating the level of peptides released by proteolysis during storage for the undigested cheese, and during digestion for the digested cheese [21].

#### 3.2.1. Peptide Profile Comparison between Undigested and Digested Cheese

As expected, the peptide profiles of the digested samples are characterized by much higher total areas (on average 997 × 10^6^, Table 3) than those of the undigested samples (219 × 10^6^, Table 2), confirming the formation of many peptides during digestion due to the breakdown of proteins by digestive enzymes. Comparing the different sections of chromatograms, it is noted that in the digested cheese there were many more peptides in the central sections (between 12 and 29 min) and much fewer in the last section after 32 min (Figure 3), while in the undigested cheese there were more peptides in the final part of the chromatogram (between 32 and 41 min, Figure 2). Consequently, the average retention time is shorter in the digested cheeses (on average 18 min) than in the undigested cheeses (on average 24 min) due to the greater presence of peptides eluted within the maximum time of 29 min in the digested samples. 

For the peptide profile before digestion (Table 2), the results report no significant differences for milk type and pasteurization level, showing that the proteolysis in the cheese was neither affected by milk storage/pre-treatment nor the level of pasteurization, and there was no interaction among the factors as reported in a previous work [14]. On the other hand, there was an increase in proteolysis during the storage of the cheese to which proteolytic enzymes of different origins may have contributed, such as plasmin, the residual rennet enzyme, and starter and non-starter lactic bacteria enzymes [24].

#### 3.2.2. Changes in Peptide Profile Due to Heat Treatments

The peptide profile of the digested cheese allowed us to monitor the digestion of proteins by reporting the conversion of proteins into peptides. The peptide profile was detected from digested samples obtained after the duodenal phase to show a fingerprint of the digestion endpoint and to evaluate if there were differences in the peptide profile due to the treatments of milk used in the cheesemaking. The peptide profile detection method appears to be an adequate qualitative method to show the results of protein hydrolysis after gastrointestinal simulation, as previously demonstrated by Parrot et al. [21]. 

As shown in Table 3, milk pre-treatment significantly affected the peptide profile after cheese digestion. The mean retention times were significantly reduced from 18.92 min for F milk to 17.39 min for P milk, and this decrease is related to the increase in the seg1% and the reduction in the seg4%. Moreover, the results show that pasteurization level also impacts the modification of peptide elution profiles obtained after cheese digestion. Strongly pasteurized milk cheese is characterized by a lower total area compared to minimally pasteurized milk cheese. Significant differences were observed in the area % values of segments 1 and 2 between the cheeses obtained from strongly pasteurized milk and minimally pasteurized milk (Table 3). The comparison between the chromatograms for the storage time showed a shorter mean retention time in the digested cheese at 21 days of storage than at 4 days of storage due to higher segment 1 and 2% values of the chromatogram (Table 3). 

**Figure 4:**
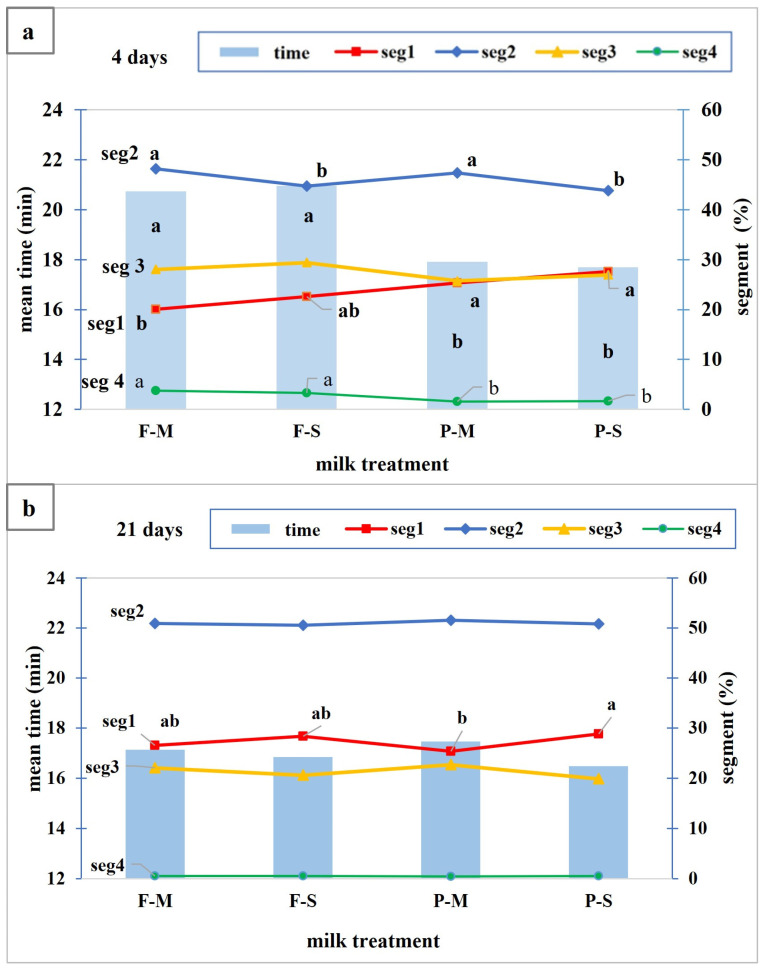
Mean retention time and segments of peptide profile in digested cheese considering the data of 4-day-aged cheese (**a**) separately from data of 21-day-aged cheese (**b**). F-M: fresh minimally pasteurized milk; F-S: fresh strongly pasteurized milk; P-M: pre-treated minimally pasteurized milk; P-S: pre-treated strongly pasteurized milk. For each parameter, different letters in the graph indicate significant differences (*p* < 0.05).

Moreover, we found an interaction among the three factors (milk*storage and pasteurization level*storage, Table 3) and therefore, the parameters of the peptide profile (mean retention time and segment % values) are reported in Figure 4 considering the data of the 4-day-aged cheese separately from the data of the 21-day-aged cheese. These interactions mean that there was a different effect due to the pre-treatment and pasteurization level in the 4-day-aged cheese compared to the 21-day-aged cheese.

The peptide profile of the digested cheese was affected by milk pre-treatment after 4 days of storage; indeed, the mean retention time was significantly higher in the F-M and F-S cheeses than in the P-M and P-S cheeses and this was correlated to a higher seg4 % and a lower seg1% (Figure 4a). On the contrary, in the 21-day-aged cheese (Figure 4b), this effect was not observed; these significant differences disappeared and only seg1 resulted in being significantly higher in the P-S cheese than in the P-M cheese, showing the effect of S or M pasteurization only in cheese from milk subjected to preliminary heat treatment.

#### 3.2.3. Peptide Profile Determination to Study Protein Digestion In Vitro

In vitro digestion, simulating the gastrointestinal digestion of proteins and peptides, investigates the effect of digestive enzymes on the peptide profile of cheese. The caseins, in particular αs1- and β-caseins which represent 36 and 30% of bovine milk caseins, respectively, with a molecular mass of about 24.0 kDa, are rapidly hydrolyzed in the gastric phase by pepsin (at pH 2) into large peptides, as reported by Parrot et al. [21]. After subsequent hydrolysis by pancreatin in the duodenal phase, proteins and large peptides of the peptic digest are degraded further into fragments of lower molecular mass.

The use of the peptide profile detected by HPLC to monitor protein digestion was previously presented by Matar et al. [25], who also analyzed the effect of milk fermentation by Lactobacillus helveticus on the modification of protein and peptide elution profiles obtained after digestion to study the changes induced by the action of digestive enzymes on milk protein. 

The retention time of small peptides seems to depend mainly on amino acid composition and molecular weight (up to 15 residues), while the retention time of larger peptides and proteins is also influenced by other factors such as their conformational structure [26]. The breakdown of each peptide bond by digestive enzymes leads to the release of two peptide fragments with the exposure of two new hydrophilic chemical groups in the solution, resulting in both a reduction in the molecular weight distribution of the peptides in the mixture and a reduction in the hydrophobicity of the smaller peptides.

Therefore, the longer retention time of peptides might be caused by both their hydrophobicity and their higher molecular weight, and as expected, the hydrolysis of proteins reduced the mean retention time of the peptide profile. The use of the mean retention time corresponding to the overall distribution of peptides synthetically represents the effect of digestive enzymes on the hydrolysis of cheese proteins. It is assumed that a shorter mean retention time could indicate the greater digestibility of a cheese as it is characterized after digestion by peptides that are on average more hydrophilic and/or smaller [21].

Considering the effects of heat treatments on the milk protein denaturation and aggregation reported in numerous studies [2], we hypothesize that these changes in the protein structure of milk used in cheesemaking may affect the hydrolysis of the cheese proteins during digestion. 

### 3.3. Amino Acid Content after In Vitro Digestion

The content of AAs was determined from the cheeses after in vitro digestion (Table 4). From the data obtained, it is possible to note that there was a significantly higher content of total AAs in the cheese produced from milk subjected to a higher temperature of pasteurization and, as expected, in the cheese after 21 days of storage No significant difference was found between cheese from fresh and pre-treated milk for the content of total AAs and individual AAs released after digestion (Table 4).

The increase in the AA content at the end of the in vitro digestion could be an indication of the greater protein digestibility of the cheese because the final obtained sample contained the AA produced by the activity of the digestive enzymes, and therefore, proteins are more hydrolyzed when there is a higher AA content in the digested sample [27].

#### 3.3.1. Determining Total AA Content to Assess In Vitro Digestion

Protein digestion is defined as the enzymatic breakdown of proteins into their components for obtaining molecules available for absorption. This dynamic physiological event is influenced by both gastrointestinal conditions and the physical and chemical characteristics of the food matrix. After ingestion, cheese is digested in the gastrointestinal tract, and milk proteins are further degraded to provide free AAs that can be adsorbed at the intestinal level [28]. 

Moreover, it should be highlighted that not only free amino acids but also dipeptides and tripeptides are bioavailable for intestinal adsorption. It is known that not all proteins are degraded to free AAs and are adsorbable as individual AAs but a fraction of the total ingested proteins are absorbed as peptides [29].

A great variety of in vitro digestion tests are described in the literature, all trying to resemble physiological conditions in humans. This is due to the difficulties in the evaluation of digestion in vivo. Moreover, the amount of released AAs after in vitro digestion is not comparable to that in an in vivo situation because the absorption processes are not present in vitro models, lacking intestinal barrier activity with a micro-villous border able to further protein hydrolysis and the absorption of digested end products [27].

Static digestion models, such as the one used in this work, aim to simplify a highly complex and dynamic physiological process, not considering peristalsis and gastric emptying, which have a great influence on food breakdown in in vivo digestion [19].

However, Egger et al. [27] reported that the total free AA content in digested milk was similar at the end of the intestinal phase, comparing static and dynamic in vitro systems, although the kinetics of protein hydrolysis, especially during the gastric phase, were better represented in the dynamic in vitro protocol, due to the constant addition of active enzymes. Hence, dynamic in vitro digestion is more highly indicated to monitor the kinetics of gastric and duodenal digestion, but static digestion is a useful method to study the endpoint of digestion.

#### 3.3.2. Individual AAs Released after Digestion

The increase in total AA content in the cheese from the S pasteurized milk was due to a significant increase in the individual AA content of Asp, Ser, Arg, Thr, Ala, Tyr Met and Phe (Table 4). Among the different AAs, Arg, Tyr, Lys, Leu and Phe were also present in the highest amount in the digested cheese.

The most abundant AA detected in digested cheese was Arg, which is known as one of the major components of milk proteins. Generally, the bioaccessible AAs seem to represent the AA composition of dairy proteins (except acid AAs), and in particular, the concentration of basic AAs tended to be similar to basic AA concentration found in the dairy proteins of milk. However, the very low proportion of the acid AAs (Asp and Glu) measured in the digested samples is inconsistent with their content in milk proteins [4].

Moreover, the basic AAs, Arg and Lys, found in a high proportion in the digested cheese, are the two AA targets of trypsin and carboxypeptidase B [4]. The release of free AAs at the end of duodenal digestion could depend even on the specificity of proteases (chymotrypsin and trypsin) and peptidases (aminopeptidases and carboxypeptidases) contained in pancreatin.

In the digested samples, there was also a high concentration of Leu, a hydrophobic AA representing about 9% of the AAs in milk proteins and reported by Rinaldi et al. [4] as one of the most important bioaccessible AAs at the end of the duodenal digestion. Furthermore, Kopf-Bolanz et al. [30] showed that Leu and Phe were the most abundant free AAs measured in pasteurized milk at the end of duodenal digestion, which is in agreement with the high release of these AAs also found in our digested samples. 

Bonds following the aromatic amino acid Phe in a peptide sequence are very susceptible to hydrolysis by pepsin in gastric digestion, and Phe release increases considerably after the duodenal phase due to the combined action of chymotrypsin and exopeptidases contained in pancreatin. The release of Tyr, another aromatic AA, increases too with hydrolysis due to pancreatin. α-Chymotrypsin, which is an endopeptidase present in pancreatin, has long been known to hydrolyze the proteins liberating peptides with an aromatic AA at their C-terminal extremity [21,31]. Phe and Tyr could then be released by the subsequent action of carboxypeptidase A. Most neutral AAs were detected in low concentrations in the digested samples, which is in agreement with what was reported by Kopf-Bolanz et al. [30]. 

Moreover, it seems that both the microstructure of dairy products and the heat treatment applied to milk may induce a difference in the individual AAs released upon duodenal digestion, as reported in previous studies [4].

### 3.4. Influence of Heat Treatment and Microstructure on Protein Digestion

The obtained results showed an increase in the protein digestion of cheese due to the higher pasteurization level of milk assessed by both the peptide profile and AA content. The greater digestibility of the cheese produced from highly pasteurized milk was probably due to physico-chemical and structural modifications induced in the proteins during heat treatment. However, due to the interactions between the food matrix and the heat-induced physico-chemical modifications, it is difficult to understand the influence of heat treatment on the digestibility of milk proteins because the findings are affected by the in vitro or in vivo methods used to monitor digestion [27].

In accordance with our results, other authors suggested a higher pepsin susceptibility of caseins depending on the severity of the heat treatment applied to milk, reporting that caseins were degraded more rapidly in sterilized milk (120 °C/30 s) than in pasteurized (82 °C/30 s) or raw milk during gastric digestion [32]. Moreover, protein digestibility can be affected by cheese processing and the time of ripening [19]. 

Structural and functional modifications of milk proteins caused by heating are well-known, but the impact of heat treatments on the digestion of milk proteins is not yet fully explained. Mulet-Cabero et al. [6], comparing pasteurized (72 °C for 15 s) and UHT (140 °C for 3 s) milk, reported the formation of a less compact clot during gastric digestion in vitro, due to the higher temperature of the treatment. 

Moreover, whey proteins are denatured by temperatures above 75 °C. In particular, native β-Lg is resistant to pepsin digestion while heating β-Lg unfolds and exposes hydrophobic groups with aggregate generation, which show greater susceptibility to proteolysis [6]. Our results are therefore in agreement with the previously published studies showing that protein digestion increases with an increase in pasteurization temperature probably due to the denaturation of whey proteins, the unfolding of the globular molecules and aggregate generation.

However, heat treatment induced aggregation between caseins and whey proteins but its effect on coagulation and digestion behavior is controversial. Aggregation induced by heat treatment might limit or modify the accessibility to some cleavage sites by digestive enzymes, which might affect the peptides released during digestion [28]. Indeed, casein digestion appears to be affected by matrix structure and processing, and digestion results depend on the applied in vitro protocol [27]. 

Cheese digestibility is different from that of milk and individual caseins or whey proteins; although the chemical composition of the protein is the same, the cheese structure is more complex. Cheese microstructure and texture have been shown to influence the rate of milk protein hydrolysis [33,34]. During cheese making, coagulation of the casein micelles is achieved by the enzymatic (chymosin) hydrolysis of k-casein and caseins, which are heat stable both as casein micelles and in the form of caseinates. Milk used for cheese production is normally heat-treated, inducing the denaturation of whey proteins and their incorporation into the cheese curd. Barbé et al. [8] compared the in vivo digestion of milk and rennet gels made from milk heated in the same condition (90 °C/10 min). The absorption of AAs was delayed and reduced due to rennet gel feeding, confirming the importance of a coagulated rennet matrix and heat treatment in protein digestion even in in vivo conditions.

### 3.5. Principal Component Analysis

A principal component analysis (PCA) was performed to obtain an overview and a classification of the cheese according to the considered factors. The parameters considered for a total of 29 variables were the physico-chemical characteristics of the cheese, the parameters of the peptide profile in the digested cheese and the content of individual and total AAs (previously reported in Table 1, Table 3 and Table 4, respectively). The first three variables extracted (PC1-PC3) jointly explained almost 60% of the total variability.

**Figure 5:**
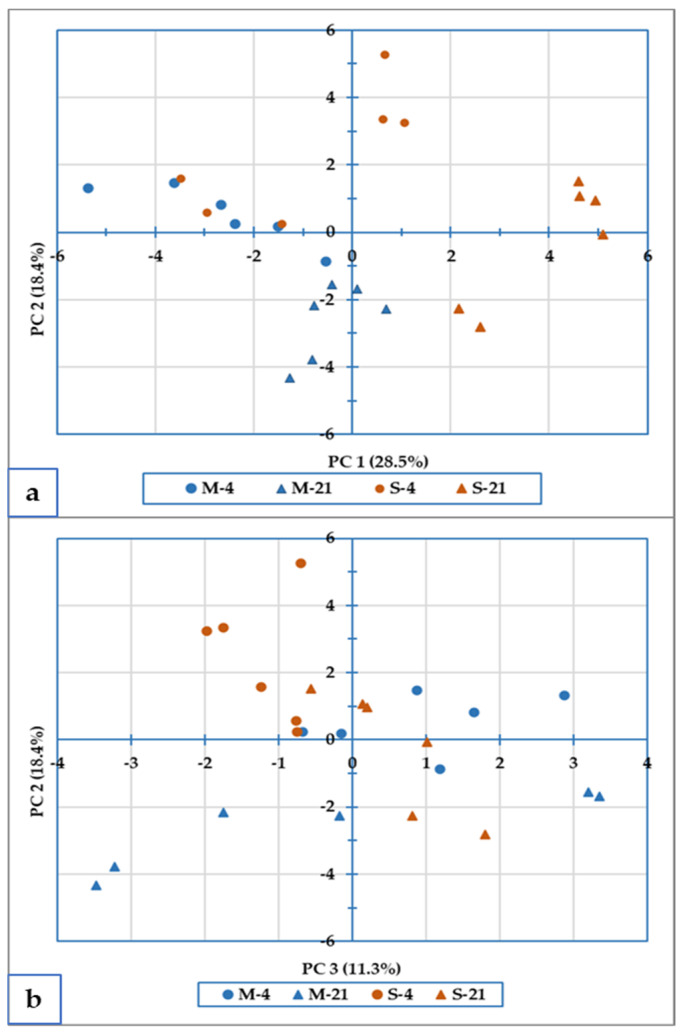
Biplot of the PCA considering the level of milk pasteurization (M and S) and cheese storage (4 and 21 days); (**a**) scatterplot in the PC1*PC2 space, and (**b**) scatterplot in the PC2*PC3 space. The used variables are reported in Table 1, Table 3 and Table 4 for a total of 29 variables. M-4: 4-day-aged cheese from minimally pasteurized milk; M-21: 21-day-aged cheese from minimally pasteurized milk; S-4: 4-day-aged cheese from strongly pasteurized milk; M-21: 21-day-aged cheese from strongly pasteurized milk.

In the scatterplot in the PC1*PC2 space (Figure 5a), the cheese samples are labeled considering the two levels of milk pasteurization used to make cheese (M and S are represented by blue and orange, respectively), and cheese storage time (4 and 21 days, as represented by dots and triangles, respectively, in Figure 5). It is possible to observe that samples of cheese made from minimally pasteurized milk are positioned mainly on the negative side of the PC1 axis (represented in blue, on the left part of the graph), while cheese from strongly pasteurized milk is mainly positioned on the positive PC1 axis (in orange on the right part of the graph, Figure 5).

On the second axis (PC2), the 4-day-aged cheese is separated from the 21-day-aged cheese. Indeed, the samples of cheese after 4 days of storage are mainly positioned on the positive side of the PC2 axis (pictured as dots in the upper part of the graph) while samples after 21 days of storage are positioned on the negative PC2 axis (pictured as triangles in the lower part of the graph).

This multivariate approach allowed an analysis of variance of many variables jointly, without making a priori assumptions about the nature of the analyzed samples. The sample clustering, in agreement with the tested experimental theses, underlines the presence of unobservable (co)variance structures, showing that there is a dependency structure between the n observations. Cluster separation can be more marked as in the case of the M-21 and S-21 theses, or more obscure as in the case of theses M-4 and S-4 theses, which tend to partially overlap (Figure 5a).

The sample scatterplot in the PC2*PC3 space (Figure 5b) shows the separation according to the cheese storage time along PC2 (4 and 21 days, represented by dots and triangles, respectively), whereas along the third component (PC3), it is not possible to observe any form of sample clustering that is in agreement with the tested experimental theses. However, along the PC3 axis, it is possible to appreciate a greater variability of the minimally pasteurized samples (represented in blue), compared to strongly pasteurized ones (in orange).

**Figure 6:**
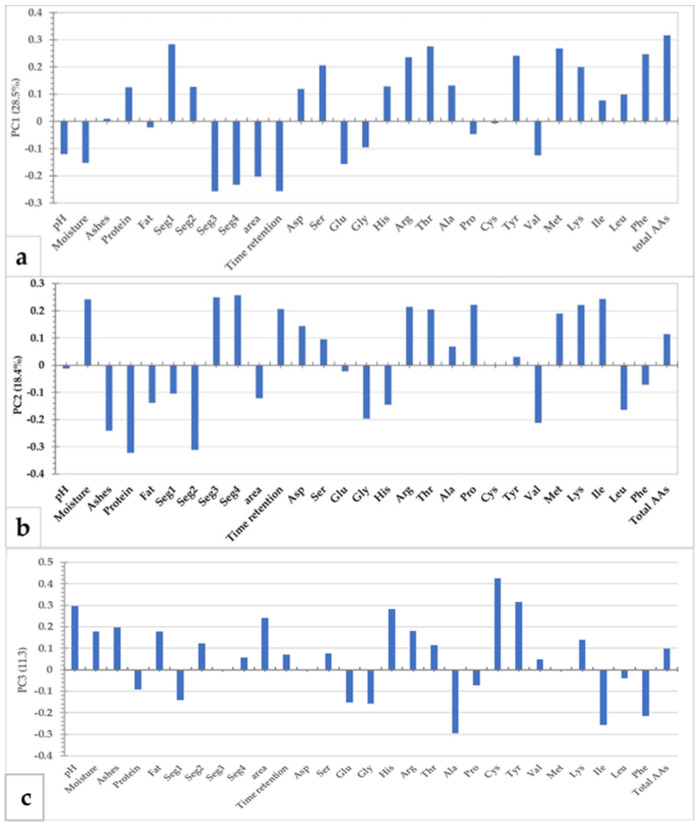
(**a**) Graph of the loadings of PC1, (**b**) graph of the loadings of PC2, and (**c**) graph of the loadings of PC3. The used variables are reported in Table 1, Table 3 and Table 4 for a total of 29 variables.

Considering the contribution of the individual variables (loadings reported in Figure 6), PC1 is more positively related to seg1, individual AAs—Arg, Thr, Tyr, Met, and Phe—and total AAs while it is inversely related with seg3, seg4, area and retention time. PC2 is more positively related to moisture, seg3, seg4, Pro, Lys and Ile while it is inversely related to ashes, protein, seg2 and Val (Figure 6b), whereas the greater variability found for the minimally pasteurized samples (M) is attributable to the pH and the content of His, Ala, Cys e and, to a lesser amount, Ile and Phe (Figure 6c). The PCA analysis allowed us to discriminate between the cheese from different levels of milk pasteurization and the cheese with different storage periods, confirming the previously described results.

## 4. Conclusions

In conclusion, the peptide profile after in vitro digestion highlighted the different effects of heat pre-treatment and pasteurization levels on the produced cheeses. However, the effect of the milk pre-treatment on the digested peptide profile changed according to the cheese ripening time. The protein digestibility of the cheese after 4 days of aging was positively affected by the use of pre-treated milk. In the longer-aged cheese, no differences were found between fresh and pre-treated milk. This was probably due to the greater degree of proteolysis found in the longer-aged cheese which may have made the effect less evident. The higher content of AAs found in the digested cheese from strongly pasteurized milk could suggest higher digestibility due to the higher pasteurization temperature.

These effects on the in vitro digestion of cheese could depend on heat treatments used for milk which cause the denaturation of proteins, in turn favoring proteolysis. From these results emerges the importance of the management of heat treatments on the digestion of proteins in soft cheese. Further studies are needed to understand the mechanisms by which heat treatments affect protein digestibility, also considering the effect of the cheese matrix in its complexity and the differences between cheese types.

## Figures and Tables

**Table 1:** Estimated least square means of physico-chemical characteristics of the cheeses considering three fixed factors with two levels each: type of milk (F and P), pasteurization level (M and S), and cheese storage (4 days and 21 days), also considering their interactions. F: fresh milk; P: pre-treated milk; M: minimal pasteurization; S: strong pasteurization.

Parameter	Milk		Pasteurization		Storage		Interaction			
	F	P	M	S	4 Days	21 Days	m*p	m*s	p*s	m*p*s
pH	5.36 ± 0.52	5.19 ± 0.21	5.40 ^a^ ± 0.53	5.15 ^b^ ± 0.13	5.29 ± 0.40	5.27 ± 0.42	ns	ns	ns	ns
Moisture (%)	58.66 ± 2.74	58.44 ± 2.18	58.35 ± 3.05	58.75 ± 1.71	60.08 ^a^ ± 1.99	57.02 ^b^ ± 1.85	0.02	ns	ns	ns
Ash (%)	2.77 ± 0.40	2.78 ± 0.28	2.80 ± 0.32	2.76 ± 0.37	2.63 ^b^ ± 0.29	2.92 ^a^ ± 0.33	ns	ns	ns	ns
Protein (%)	18.40 ^b^ ± 0.63	18.79 ^a^ ± 0.64	18.80 ^a^ ± 0.73	18.39 ^b^ ± 0.52	18.18 ^b^ ± 0.58	19.01 ^a^ ± 0.45	0.002	ns	ns	ns
Fat (%)	21.21 ± 1.31	20.53 ± 1.61	21.31 ^a^ ± 1.79	20.43 ^b^ ± 0.96	20.40 ^b^ ± 1.23	21.33 ^a^ ± 1.61	ns	ns	ns	ns

Data are expressed as least square means ± standard deviation. Means followed by a different superscript letter in the row for each factor are significantly different (*p* < 0.05). ns: not significant. Interactions: m*p (milk*pasteurization), m*s (milk*storage), p*s (pasteurization*storage), and m*p*s (milk*pasteurization*storage).

**Table 2:** Estimated least square means of the area % of each segment (seg), the total area, and the mean retention time of the peptide profile in the undigested cheese. Three factors with two levels each were considered: type of milk (F and P), pasteurization level (M and S), and cheese storage (4 days and 21 days) as well as their interactions. F: fresh milk; P: pre-treated milk; M: minimal pasteurization; S: strong pasteurization.

Parameters		Milk		Pasteurization		Storage		Interaction			
		F	P	M	S	4 Days	21 Days	m*p	m*s	p*s	m*p*s
%	Min										
seg1	1–12	23.68 ± 7.09	23.69 ± 6.16	23.82 ± 6.34	23.55 ± 6.93	29.80 ^a^ ± 1.43	17.56 ^b^ ± 0.75	ns	ns	ns	ns
seg2	12–24	21.95 ± 2.27	21.21 ± 2.68	21.05 ± 2.84	22.11 ± 1.98	19.60 ^b^ ± 1.78	23.56 ^a^ ± 0.72	ns	ns	ns	ns
seg3	24–36	34.36 ± 7.21	34.04 ± 7.72	34.59 ± 6.42	33.8 ± 8.37	27.36 ^b^ ± 2.02	41.04 ^a^ ± 0.80	ns	ns	ns	ns
seg4	36–42	20.02 ± 2.23	21.07 ± 3.84	20.54 ± 3.18	20.55 ± 3.20	23.24 ^a^ ± 1.89	17.86 ^b^ ± 0.49	ns	ns	ns	ns
total area (10^6^)		224.3 ± 60.1	205.3 ± 58.3	219.5 ± 67.9	210.0 ± 50.6	163.8 ^b^ ± 22.6	265.8 ^a^ ± 27.7	ns	ns	ns	ns
mean retention time (min)		23.88 ± 1.50	24.18 ± 1.00	23.95 ± 1.11	24.10 ± 1.43	22.94 ± 0.60	25.11 ± 0.50	ns	ns	ns	ns

Data are expressed as least square means ± standard deviation. Means followed by a different superscript letter in the row for each factor are significantly different (*p* < 0.05). ns: not significant. Interactions: m*p (milk*pasteurization), m*s (milk*storage), p*s (pasteurization*storage), and m*p*s (milk*pasteurization*storage).

**Table 3:** Estimated least square means of the area % of each segment (seg), the total area, and the mean retention time of the peptide profile in the digested cheese. Three fixed factors with two levels each were considered: type of milk (F and P), pasteurization level (M and S), and cheese storage (4 days and 21 days) as well as their interactions. F: fresh milk; P: pre-treated milk; M: minimal pasteurization; S: strong pasteurization.

Parameters		Milk		Pasteurization		Storage		Interaction			
		F	P	M	S	4 Days	21 Days	m*p	m*s	p*s	m*p*s
%	min										
seg1	1–12	24.37 ^b^ ± 3.55	26.79 ^a^ ± 1.79	24.33 ^b^ ± 2.81	26.83 ^a^ ± 2.78	23.90 ^b^ ± 3.16	27.25 ^a^ ± 1.67	ns	0.001	ns	ns
seg2	12–24	48.60 ± 2.68	48.39 ± 3.34	49.49 ^a^ ± 2.00	47.49 ^b^ ± 3.48	46.03 ^b^ ± 2.03	50.95 ^a^ ± 0.62	ns	ns	0.007	ns
seg3	24–36	25.01 ± 4.10	23.80 ± 3.10	24.62 ± 2.79	24.19 ± 4.40	27.52 ^a^ ± 1.66	21.29 ^b^ ± 1.53	ns	ns	0.02	ns
seg4	36–42	2.03 ^a^ ± 1.64	1.02 ^b^ ± 0.64	1.56 ± 1.46	1.49 ± 1.24	2.55 ^a^ ± 1.12	0.50 ^b^ ± 0.11	ns	0.001	ns	ns
total area (10^6^)		961 ± 247	1033 ± 242	1192 ^a^ ± 163	802 ^b^ ± 93	1036 ± 244	958 ± 244	ns	ns	ns	ns
mean retention time (min)		18.92 ^a^ ± 2.12	17.39 ^b^ ± 0.77	18.31 ± 1.59	17.99 ± 1.96	19.33 ^a^ ± 1.66	16.98 ^b^ ± 0.53	ns	0.002	ns	ns

Data are expressed as least square means ± standard deviation. Means followed by a different superscript letter in the row for each factor are significantly different (*p* < 0.05). ns: not significant. Interactions: m*p (milk*pasteurization), m*s (milk*storage), p*s (pasteurization*storage), and m*p*s (milk*pasteurization*storage).

**Table 4:** Estimated least square means of amino acid (AA) content after in vitro digestion of cheese considering three fixed factors with two levels each: type of milk (F and P), pasteurization level (M and S), and cheese storage (4 days and 21 days) as well as their interactions. F: fresh milk; P: pre-treated milk; M: minimal pasteurization; S: strong pasteurization.

mg/100 g	Milk		Pasteurization		Storage		Interaction			
	F	P	M	S	4 Days	21 Days	m*p	m*s	p*s	m*p*s
Asp	14.5 ± 3.1	14.0 ± 1.9	13.1 ^b^ ± 2.3	15.4 ^a^ ± 2.3	14.1 ± 2.2	14.4 ± 2.9	0.013	ns	ns	ns
Ser	50.4 ± 10.9	49.3 ± 6.8	45.1 ^b^ ± 5.4	54.7 ^a^ ± 9.3	47.4 ± 5.1	52.3 ± 11.3	ns	ns	0.014	ns
Glu	54.6 ± 7.7	54.0 ± 5.9	55.4 ± 6.7	53.3 ± 6.9	56.0 ± 7.2	52.6 ± 6.1	ns	ns	ns	ns
Gly	178.8 ± 30.3	182.2 ± 20.5	185.3 ± 19.4	175.7 ± 30.3	180.3 ± 24.5	180.7 ± 27.3	ns	ns	ns	ns
His	37.2 ± 9.9	36.4 ± 9.9	33.8 ± 6.2	39.8 ± 11.7	33.2 ± 8.8	40.4 ± 9.5	ns	ns	ns	ns
Arg	1299.6 ± 120.2	1369.3 ± 128.0	1282.8 ^b^ ± 111.0	1386.1 ^a^ ± 123.8	1320.3 ± 94.3	1348.6 ± 155.4	ns	ns	ns	ns
Thr	134.3 ± 62.2	145.7 ± 57.5	103.2 ^b^ ± 33.0	176.8 ^a^ ± 56.7	126.3 ± 47.5	153.7 ± 67.8	ns	ns	ns	ns
Ala	124.5 ± 9.7	130.8 ± 12.4	122.1 ^b^ ± 10.5	133.1 ^a^ ± 9.7	127.0 ± 10.9	128.3 ± 12.2	ns	ns	ns	ns
Pro	221.2 ± 39.6	219.0 ± 18.6	221.6 ± 14.2	218.6 ± 41.4	229.9 ± 35.0	210.3 ± 22.0	ns	ns	ns	ns
Cys	243.0 ± 56.9	248.9 ± 48.5	258.4 ± 61.3	233.4 ± 38.7	246.1 ± 55.8	245.8 ± 49.9	ns	ns	ns	ns
Tyr	563.4 ± 106.2	564.2 ± 91.9	531.3 ^b^ ± 71.7	596.3 ^a^ ± 110.8	527.9 ^b^ ± 68.7	599.6 ^a^ ± 110.5	ns	ns	0.001	ns
Val	79.9 ± 9.1	81.4 ± 11.4	84.4 ± 8.5	76.9 ± 10.6	81.6 ± 9.87	79.7 ± 10.8	0.028	ns	ns	ns
Met	46.6 ± 19.2	55.0 ± 19.1	36.4 ^b^ ± 9.4	65.2 ^a^ ± 15.3	47.8 ± 15.1	53.8 ± 22.9	ns	ns	0.033	ns
Lys	555.8 ± 62.1	579.3 ± 45.2	548.6 ± 58.7	586.5 ± 44.4	569.6 ± 48.6	565.5 ± 61.8	ns	ns	ns	ns
Ile	202.6 ± 18.5	216.7 ± 35.8	200.7 ± 20.0	218.6 ± 34.0	215.5 ± 34.4	203.7 ± 21.7	ns	ns	ns	ns
Leu	537.0 ± 37.7	528.0 ± 43.9	528.3 ± 43.5	536.7 ± 38.3	517.8 ± 44.7	547.3 ± 30.3	ns	ns	ns	ns
Phe	966.7 ± 180.3	947.7 ± 140.9	905.7 ^b^ ± 175.5	1008.8 ^a^ ± 126.3	849.1 ^b^ ± 138.1	1065.3 ^a^ ± 89.5	ns	ns	ns	ns
Total AAs	5310.1 ± 443.6	5421.9 ± 381.5	5156.1 ^b^ ± 230.8	5575.8 ^a^ ± 446.9	5189.9 ^b^ ± 325.5	5542.1 ^a^ ± 418.9	ns	ns	ns	ns

Data are expressed as least square means ± standard deviation. Means followed by a different superscript letter in the row are significantly different (*p* < 0.05). ns: not significant. Total AAs: Σ AAs in digested samples. Interactions: m*p (milk*pasteurization), m*s (milk*storage), p*s (pasteurization*storage), and m*p*s (milk*pasteurization*storage).

## Data Availability

The data presented in this study are available upon request from the corresponding author.
